# Combined Electrochemical Deposition and Photo-Reduction to Fabricate SERS-Active Silver Substrates: Characterization and Application for Malachite Green Detection in Aquaculture Water

**DOI:** 10.3390/nano14141226

**Published:** 2024-07-19

**Authors:** Yu-Xuan Li, Yi-Ting Chen, Cheng-Tse Chang, Chao Yi (Anso) Ting, Yaumalika Arta, Mei-Yao Wu, Tsunghsueh Wu, Yu-Shen Lin, Yang-Wei Lin

**Affiliations:** 1 Department of Chemistry, National Changhua University of Education, 1 Jin-De Road, Changhua City 50007, Taiwan; m0925013@mail.ncue.edu.tw (Y.-X.L.); m1225010@mail.ncue.edu.tw (Y.-T.C.); s1125004@mail.ncue.edu.tw (C.-T.C.); yaumalikaarta@mail.ugm.ac.id (Y.A.); 2 Department of Physics, Gadjah Mada University, Yogyakarta 55281, Indonesia; 3 School of Post-Baccalaureate Chinese Medicine, China Medical University, 91, Hsueh-Shih Road, Taichung 40424, Taiwan; meiyaowu@mail.cmu.edu.tw; 4 Department of Chemistry, University of Wisconsin-Platteville, 1, University Plaza, Platteville, WI 53818-3099, USA; wut@uwplatt.edu; 5 Department of Applied Science, National Taitung University, 369, Sec. 2, University Road, Taitung 950309, Taiwan; cangeroo@nttu.edu.tw

**Keywords:** surface-enhanced Raman scattering (SERS), silver nanoparticles, electrochemical deposition, photo-reduction, malachite green, aquaculture water monitoring

## Abstract

This research introduces a novel approach using silver (Ag) nanostructures generated through electrochemical deposition and photo-reduction of Ag on fluorine-doped tin oxide glass substrates (denoted as X-Ag-Ag_y_FTO, where ‘X’ and ‘y’ represent the type of light source and number of deposited cycles, respectively) for surface-enhanced Raman spectroscopy (SERS). This study used malachite green (MG) as a Raman probe to evaluate the enhancement factors (EFs) in SERS-active substrates under varied fabrication conditions. For the substrates produced via electrochemical deposition, we determined a Raman EF of 6.15 × 10^4^ for the Ag_2_FTO substrate. In photo-reduction, the impact of reductant concentration, light source, and light exposure duration were examined on X-Ag nanoparticle formation to achieve superior Raman EFs. Under optimal conditions (9.0 mM sodium citrate, 460 nm blue-LED at 10 W for 90 min), the combination of blue-LED-reduced Ag (B-Ag) and an Ag_2_FTO substrate (denoted as B-Ag-Ag_2_FTO) exhibited the best Raman EF of 2.79 × 10^5^. This substrate enabled MG detection within a linear range of 0.1 to 1.0 µM (R^2^ = 0.98) and a detection limit of 0.02 µM. Additionally, the spiked recoveries in aquaculture water samples were between 90.0% and 110.0%, with relative standard deviations between 3.9% and 6.3%, indicating the substrate’s potential for fungicide detection in aquaculture.

## 1. Introduction

Malachite green (MG), or 4-[(4-dimethylaminophenyl)-phenyl-methyl]-N,N-dimethyl-aniline, is a synthetic triphenylmethane dye once widely used in aquaculture as an antifungal agent [1,2]. However, recent studies have raised concerns about its carcinogenic risks, particularly affecting the liver and thyroid, underscoring the need for precise detection to safeguard water and fish quality [3,4]. In China, the permissible level of MG in aquiculture animal tissue is 2 μg·kg^−1^ using an official method (national standard of PR China GB/T 19857-2005). In the EU, the use of MG for fish food was banned in 2000 [3,5]. Current detection techniques, such as mass spectrometry, offer accuracy but are limited by high costs and complexity [6,7,8]. Historically significant alternative methods, like spectrophotometry and chromatography, lack the sensitivity and specificity to detect low concentrations of MG, potentially underestimating the extent of contamination occurring in the field [9,10,11,12,13].

Surface-enhanced Raman scattering (SERS), an advancement in Raman spectroscopy, has shown promise in overcoming these limitations [14,15,16,17]. Raman signals have been enhanced through the use of surface plasmon resonance of gold (Au), silver (Ag), and copper [18,19]. By amplifying signals via electromagnetic and chemical enhancements, this non-destructive SERS method has proven effective in detecting various contaminants, including pesticides and heavy metals present in aqueous solutions [20]. Despite its potential, challenges such as complex substrate synthesis and uncertain recoveries in environmental samples remain. For instance, Xu et al. employed a solvothermal method to fabricate Fe_3_O_4_ magnetic nanospheres, which were subsequently conjugated with Au nanoparticles to create Au nanoparticle-coated Fe_3_O_4_ magnetic composite nanospheres (Fe_3_O_4_@Au MCS) [21]. Their study extended its detection capabilities beyond MG to rhodamine 6G and demonstrated a linear signal–concentration relationship for both contaminants. The wide detection range for MG was found to span from 10^−7^ to 10^−3^ M, with a detection limit (LOD) of 10^−7^ M, a level notably lower than the local water quality standards in China. These findings indicate the composite’s efficacy in detecting MG within aquaculture wastewater. Although the Fe_3_O_4_@Au MCS composites exhibit a broad detection range and applicability to actual samples, their synthesis is complex and time-consuming. Xu et al. utilized electron beam evaporation to deposit Au nanoparticles onto cicada wings, leveraging their natural, uniform three-dimensional microstructure, extensive surface area, and inherent hydrophobic properties [22]. This unique substrate facilitated the generation of numerous hotspots, enhancing the detection of prohibited aquatic pharmaceuticals such as crystal violet and MG. The detection range established for both compounds was from 10^−7^ to 10^−3^ M. Additionally, calibration lines incorporating these concentrations were successfully validated with environmental water samples. Furthermore, Dong et al. utilized Au nanoparticles as seeds to facilitate the growth of Ag on their peripheries, primarily to produce Ag nanowires and, subsequently, Ag nanocubes [23]. The synthesis of Ag nanocubes was notably time-consuming; however, through extraction filtration using filter membranes with varied pore sizes, these Ag nanocubes were efficiently isolated and adhered to silicon wafers for the enhanced detection of MG. This method, grounded in self-assembly principles, introduces MG during formation, deviating from conventional practices where MG is added after substrate synthesis. This approach potentially increases sensitivity as it allows Raman-active molecules to penetrate the interstitial spaces of the nanoparticles, thereby enhancing MG detection in the aqueous environment. The resulting detection range was found to be between 5 × 10^−7^ M and 5 × 10^−4^ M, with an LOD of 2.62 × 10^−7^ M. Meanwhile, Qu et al. deposited Au and Ag alloy nanoparticles onto paper substrates using a spray technique [24] to produce a flexible Raman substrate capable of detecting contaminants in fish samples directly without the need for fish meat removal. Demonstrating high sensitivity toward MG, methylene blue, and crystal violet, the method identified MG within a detection range of 3.9 × 10^−8^ to 1.0 × 10^−5^ M and an LOD of 4.3 × 10^−9^ M. Furthermore, the recoveries of those three dyes from real samples, including water, fish meat, and fish scales, were found to be comparable to those obtained from high-performance liquid chromatography, demonstrating the paper SERS substrate as a promising, disposable diagnostic tool with portability and ease of use for field applications. Lai et al. fabricated a thin film by integrating gold nanoparticles (AuNPs) with graphene oxide (GO) on quartz substrates, leveraging the interaction between the positively charged AuNPs and negatively charged GO for effective dye adsorption [25]. This composite, denoted as AuNPs-GO, demonstrated a detection range for MG of 2.74 × 10^−11^ to 2.74 × 10^−8^ M. Remarkably, this novel material has been effectively applied to actual fish samples, achieving the lowest detection concentration of MG at 6.85 × 10^−10^ M, underscoring its significant potential in the analysis of aquatic contaminants.

In this study, we propose a combined electrochemical deposition and photo-reduction technique to fabricate Ag nanostructured substrates to refine Raman spectroscopic detection of MG in aquaculture. We aim to optimize the process of electrochemical deposition and photo-reduction to prepare Ag nanomaterials with high SERS performance on fluorine-doped tin oxide (FTO) substrates (X-Ag-Ag_y_FTO, where ‘X’ and ‘y’ represent the type of light source and the number of deposited cycles, respectively), bypassing cumbersome sample preparation to streamline and enhance environmental monitoring. By integrating advanced SERS techniques to meet aquaculture requirements, this research aims to advance sustainable practices and ensure ecological safety, marking a significant step in employing nanotechnology to resolve pressing environmental challenges.

## 2. Materials and Methods

### 2.1. Chemicals

All the analytical grade chemicals were bought from the Sigma-Aldrich company (St. Louis, MO, USA) and used without purification. Silver nitrate (AgNO₃) was used to prepare the Ag nanomaterials essential for the SERS-active substrates. Potassium nitrate (KNO₃) was used in the supporting electrolyte solution for the electrochemical deposition. Sodium citrate tribasic dihydrate was used as the reducing agent in the photo-reduction to form Ag nanoparticles. FTO substrates (7 Ω, thickness: 0.7 mm) were bought from Ruilong Optoelectronics Co., Ltd. (Houlong, Miaoli County, Taiwan). MG served as the Raman reporter molecule for evaluating the enhancement factors of the SERS-active X-Ag-Ag_y_FTO substrates. Milli-Q deionized water (18.5 MΩ) was used in all experiments.

### 2.2. Characterization

The morphology of the samples was examined using a HITACHI S-4800 scanning electron microscope (SEM, HITACHI, Tokyo, Japan) operating at 30 kV for imaging and equipped with a QUANTAX Annular XFlash^®^ QUAD FQ5060 (Bruker Nano, Berlin, Germany) for energy-dispersive X-ray spectroscopy (EDS) analysis to assess elemental composition. Additionally, the prepared Ag nanostructures were detailed using a JEOL-1200EX II high-resolution transmission electron microscope (TEM, JEOL, Tokyo, Japan) at an accelerating voltage of 120 kV. The optical properties were evaluated using an Evolution 200 UV-Vis spectrometer (Thermo Fisher, New York, NY, USA). Raman spectroscopic analysis was conducted using a confocal micro-Raman system with a 20 mW 780 nm laser (Thermo Scientific Inc., New York, NY, USA).

### 2.3. Electrochemical Deposition of Ag_y_FTO Substrate

Electrochemical deposition is driven by an electric field, which induces ion migration and redox reactions at the electrodes. This process involves electron transfer at the electrode surface, leading to the reduction of metal ions at the cathode and the formation of a metal coating on the conductive surface. This method generates a nanostructure on the electrode surface, suitable for use as a Raman substrate [26,27]. FTO substrates are particularly advantageous due to their conductivity, which allows for the straightforward electrodeposition of metallic nanostructures such as silver or gold. Since a metal layer was deposited on the surface of the FTO substrate, no energy damage occurred when the laser light source irradiated the surface during Raman spectrum measurement. This study employed the FTO substrate as the working electrode for electrochemical deposition. Before use, the FTO substrate was thoroughly cleaned with a neutral detergent, followed by rinsing with acetone and ethanol to remove any residues. The substrate was then rinsed with deionized water to eliminate any remaining solvents and dried in an oven.

**Scheme 1:**
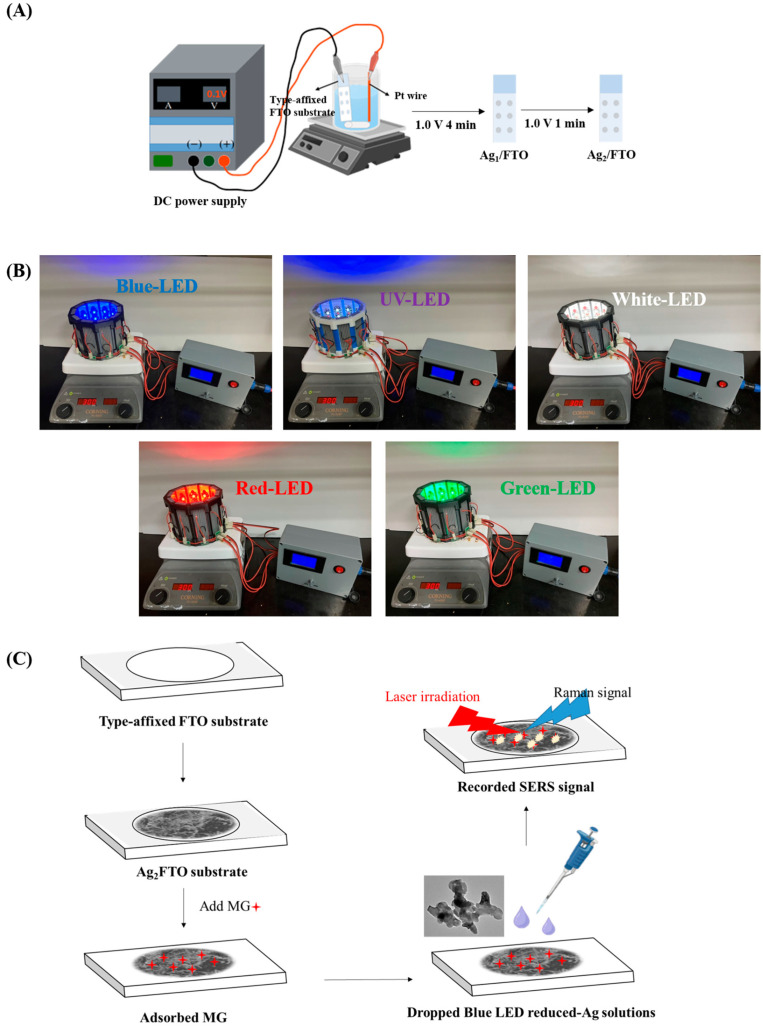
(**A**) Electroplating system setup, (**B**) light irradiation system for prepared X-Ag, and (**C**) schematic illustration of nanoparticle-on-mirror (NPoM) fabrication for SERS analysis.

To create small-area Ag nanostructures on FTO, tape with a 2 mm hole was affixed to the cleaned FTO substrate. An electrolyte concentration of 0.1 M KNO₃ and 5.0 mM AgNO₃ was prepared by dissolving 0.2 g of KNO₃ in 19.8 mL of deionized water, to which 0.2 mL of 0.5 M AgNO₃ solution was added. The FTO substrate, covered with hole-punched tape, was submerged in this electrolyte solution and subjected to electroplating using a platinum electrode as the counter electrode, applying a voltage of 0.8–1.5 V for 1–5 min to study the effect of potential and deposition duration (the distance between two electrodes was 1 cm, as shown in Scheme 1A). The Ag-coated FTO substrates were rinsed with deionized water, and the tape was removed before they were dried in an oven. The resulting Ag nanomaterial from the first cycle of electrochemical deposition was designated as the Ag_1_FTO substrate. The second cycle, Ag_2_FTO substrate, was formed using the same electrolyte composition via electrochemical deposition at 0.8–1.5 V for 0.5–5 min.

### 2.4. Photo-Reduction of X-Ag Nanoparticles

As the second component for SERS enhancement, X-Ag nanoparticles were synthesized through LED light-induced chemical reduction. A solution containing 0.23 g of sodium citrate dissolved in 1.0 mL deionized water was then transferred into a 1.5 mL vial. Next, 49.0 mL of deionized water was added to a quartz glass bottle, followed by the addition of 0.5 mL each of the prepared sodium citrate solution and AgNO₃ solution to attain concentrations of 9.0 mM for sodium citrate and 0.1 mM for AgNO₃, respectively. Then, each solution was placed in a light irradiation system (Kaishin Co., Ltd., New Taipei City, Taiwan) and subjected to blue (B, 460 ± 5 nm), ultraviolet (UV, 365 ± 5 nm), white (W, 6500–7000 K), green (G, 530 ± 5 nm), and red (R, 620 ± 10 nm) LED lights, each set at 10 W with varied irradiation times from 30 to 150 min (as shown in Scheme 1B). This process yielded X-Ag nanoparticles synthesized under different conditions labeled as B-Ag, UV-Ag, W-Ag, G-Ag, and R-Ag, corresponding to the LED light color used during synthesis.

### 2.5. MG Detection by SERS-Active X-Ag-Ag_y_FTO Substrate

This study selected MG as the Raman probe molecule to characterize newly created SERS substrates, which have so-called nanoparticle-on-mirror (NPoM) configurations for SERS analysis, as depicted in Scheme 1C. First, 1.0 µL of MG solution was applied to the Ag_y_FTO substrate. Then, X-Ag nanoparticles were concentrated by a factor of ten, and 1.0 µL of this concentrated X-Ag solution was added to the Ag_y_FTO surface. Each Raman spectrum was collected using a 20 mW 780 nm laser with a signal accumulation duration of 1 s and 10 scans.

The Raman enhancement factor (*EF*) using our *SERS* substrates was calculated as follows:

$$EF=\frac{I_{SERS}}{C_{SERS}}\times \frac{C_{Raman}}{I_{Raman}}$$

In this formula, *I_SERS_* and *I_Raman_* denote the spectral intensities of MG at the wavenumber 1614 cm^−1^ for the SERS-active X-Ag-Ag_y_FTO and an FTO substrate, respectively. *C_SERS_* and *C_Raman_* represent the concentrations used in the Raman measurements. For the Raman signal of MG, an MG solution at a concentration of 0.1 M was applied to an FTO glass without any silver deposition. Similarly, the SERS signal was generated from an MG solution at a concentration of 1.0 µM added to the Ag_y_FTO and X-Ag-Ag_y_FTO substrates.

## 3. Results and Discussion

### 3.1. Characterization and SERS Performance of Electrochemical Deposition of Ag_y_FTO Substrate

Electrochemical deposition is now a prevalent method because of its low cost and fast, straightforward procedure to produce roughened metal material on an electrode surface. Due to its high enhancement factor achieved, Ag was selected as the SERS-active metal produced by electrodeposition, and the influences of fabrication steps, such as deposition cycles, electroplating voltage, and duration, on its morphology and SERS performance were assessed in this study [28].

**Figure 1:**
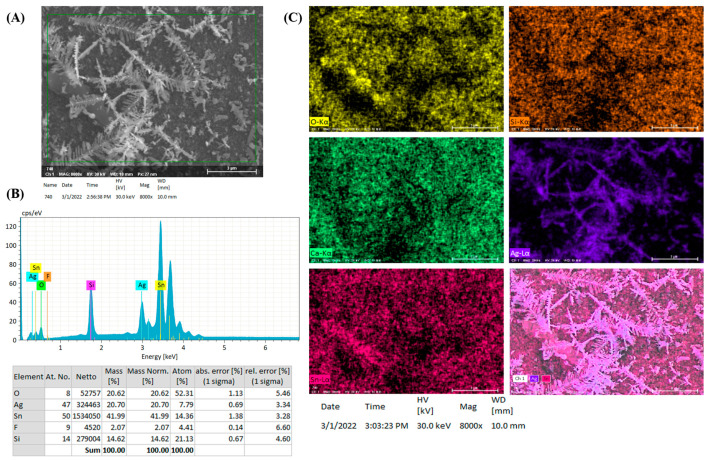
(**A**) SEM image, (**B**) EDS spectrum, and (**C**) elemental mapping of Ag_1_FTO substrate.

**Figure 2:**
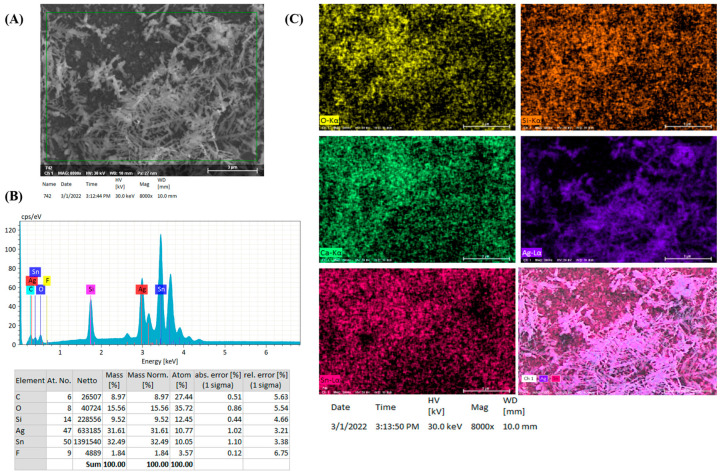
(**A**) SEM image, (**B**) EDS spectrum, and (**C**) elemental mapping of Ag_2_FTO substrate.

As surface characterization techniques, SEM and EDS analyses were conducted on the Ag_y_FTO substrate to determine the morphology and elemental composition of the Ag deposit on the FTO surface, as illustrated in Figure 1 and Figure 2. On both Ag_1_FTO and Ag_2_FTO substrates, the Ag coating exhibited a dendritic structure with a central main branch surrounded by numerous smaller branches. The dendritic structure of Ag_1_FTO appeared less dense compared to that of Ag_2_FTO. EDS and elemental mapping analysis confirmed the presence of Ag on the FTO substrate, while Sn and F, constituents of the FTO substrate, were also detected. Notably, the atomic ratio of Ag to Sn in Ag_2_FTO was approximately 1.98 times higher than in Ag_1_FTO, suggesting that more Ag was deposited with an additional deposition cycle.

**Figure 3:**
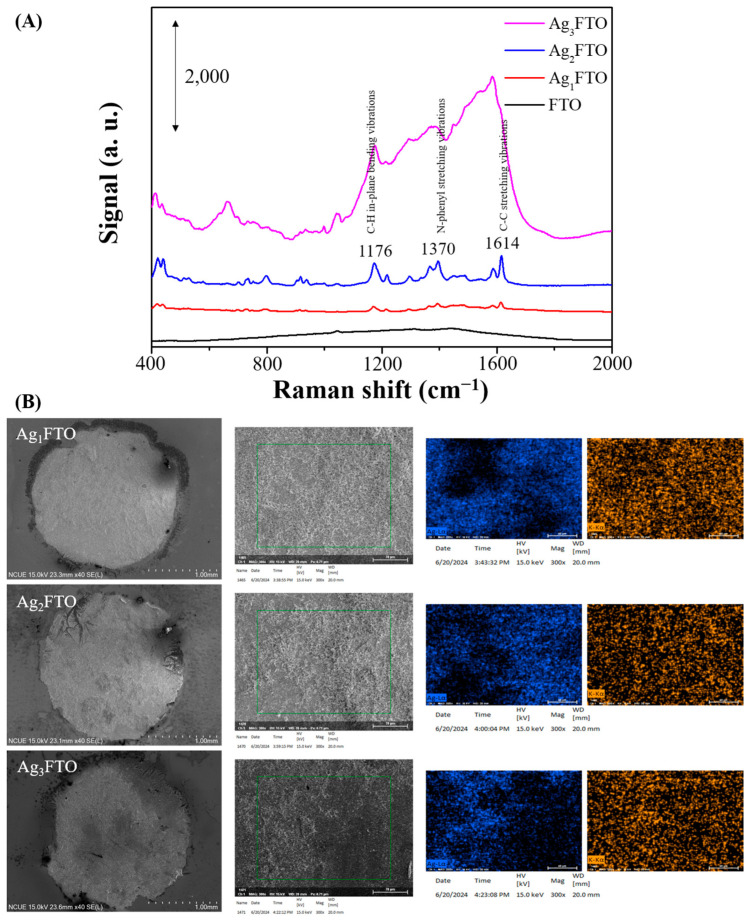
(**A**) SERS spectra of 1.0 μM MG on different substrates: FTO (black spectrum), Ag_1_FTO (red spectrum), Ag_2_FTO (blue spectrum), and Ag_3_FTO (pink spectrum). (**B**) SEM images and EDS-mapping for Ag_1_FTO, Ag_2_FTO, and Ag_3_FTO substrates.

Ag nanomaterials were electroplated onto FTO substrates with three different cycles: Ag_1_FTO (1.0 V, 4 min), Ag_2_FTO (1.0 V, 1 min/1.0 V, 1 min), and Ag_3_FTO (1.0 V, 4 min/1.0 V, 1 min/1.0 V, 1 min). Each substrate was subsequently dotted with 1 µL of 1.0 µM MG solution and left to air-dry before Raman analysis. Figure 3A illustrates that distinct Raman peaks of MG were observed: a peak at 1176 cm^−1^ corresponding to the C-H in-plane bending vibrations of the benzene ring, a peak at 1370 cm^−1^ associated with the N-phenyl stretching vibrations, and a peak at 1614 cm^−1^ attributed to the C-C stretching vibrations of the benzene ring [28,29,30]. Notably, the FTO substrate without Ag electroplating displayed no characteristic Raman peaks of MG, indicating the necessity of the Ag nanomaterials for Raman detection.

The experimental data revealed that the Raman signal intensity on the Ag_1_FTO substrate was significantly weaker than on the Ag_2_FTO substrate. This variation suggests that Ag deposition during the first electroplating process was non-uniform, resulting in less FTO surface covered with Ag. This affected the distribution and subsequent reducing signal enhancement, as shown in Figure 3B. Conversely, in the second electroplating process, areas previously free of Ag deposit were covered with Ag, increasing surface roughness and enhancing the Raman signal. This supports the hypothesis that significant surface plasmon resonance and electromagnetic field enhancement occur at the tips and edges of the Ag dendritic structure on Ag_1_FTO and Ag_2_FTO, leading to increased Raman signal strength [31,32]. However, the Ag_3_FTO substrate, prepared with three cycles of electrodeposition, exhibited a Raman signal heavily marred by background noise. This is likely due to the excessive thickness of the Ag layer on the Ag_3_FTO surface, which may lead to delamination. Delamination diminishes the number of effective Raman hot spots, resulting in a Raman signal intensity compromised by background noise. Another potential reason for the background signal observed in Figure 3A could be the presence of G- and D-bands of amorphous carbon formed via the photothermal degradation of MG. This suggests that the Ag_3_FTO substrate may have exhibited excessive localized surface plasmon resonance when excited at 780 nm.

**Figure 4:**
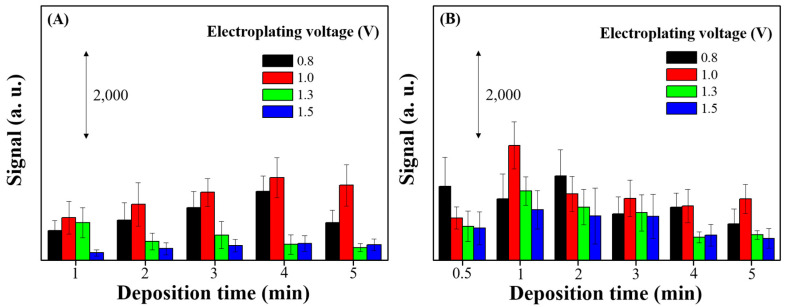
Raman signals of MG (10.0 μM) at 1614 cm^−1^ for (**A**) Ag_1_FTO and (**B**) Ag_2_FTO substrates across various electroplating voltages and deposition times.

To optimize the electroplating conditions for Ag_1_FTO and Ag_2_FTO substrates, we varied the electroplating voltage and deposition time to measure changes in the Raman signal. As indicated in Figure 4A, the Raman signal intensity (at 1614 cm^−1^) of MG under the first electroplating conditions was higher at 0.8 V and 1.0 V than at 1.3 V and 1.5 V. This suggests that lower electroplating voltage conditions promote a rougher Ag structure, enhancing the Raman signal. In addition, the optimal electroplating time was found to be 4 min. Therefore, deposition parameters (1.0 V and 4 min) were chosen as the optimal conditions for the first electrodeposition. Because the initial signal intensity on the Ag_1_FTO substrate was low, the second cycle of electrodeposition was applied to increase the SERS effect due to the increased surface roughness from the accumulation of Ag materials electroplated at different electroplating voltages (as shown in Figure 4B). Despite the higher Raman signals obtained from the substrates at higher second electroplating voltages, the large standard deviation of the Raman signal at a higher voltage suggests that deposition at 1.0 V for 1 min is more reliable as the optimal condition for Ag_2_FTO. Furthermore, from the SERS results, Ag_1_FTO had an EF of 4.52 × 10^4^, lower than that of Ag_2_FTO with an EF of 6.15 × 10^4^, as detailed in Table 1, suggesting that the Ag_2_FTO substrate is more effective for further study.

**Figure 5:**
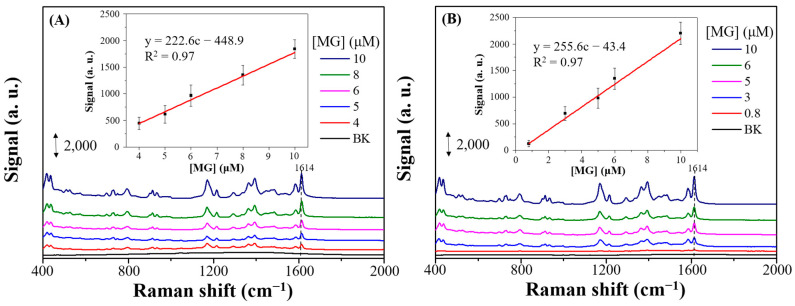
SERS spectra (Inset: Raman signal at 1614 cm^−1^) for varying concentrations of MG using (**A**) Ag_1_FTO and (**B**) Ag_2_FTO substrates.

The sensitivity study on MG detection from optimized Ag_1_FTO and Ag_2_FTO substrates is detailed in Figure 5. After adding solutions with varying concentrations of MG, the SERS signal at 1614 cm^−1^ was used to generate a calibration curve. The results revealed that Ag_1_FTO detected MG within a linear range of 4 µM–10 µM, with an LOD of 0.52 µM at an S/N ratio of 3.0 ($LOD=\frac{3\sigma_{blank}}{slope}=\frac{3\times 38.5}{222.6}=0.52\;\mu M$) and an R^2^ value of 0.97 (Inset in Figure 5A). In contrast, Ag_2_FTO exhibited a broader linear range of 0.8 µM to 10 µM, a lower LOD of 0.21 µM, an S/N ratio of 3.0 ($LOD=\frac{3\sigma_{blank}}{slope}=\frac{3\times 17.9}{255.6}=0.21\;\mu M$), and an R^2^ value of 0.97 (Inset in Figure 5B), indicating that Ag_2_FTO was about 2.47 times more sensitive than Ag_1_FTO. This increased sensitivity is likely due to the denser Ag nanomaterial of Ag_2_FTO, enhancing MG detection at lower concentrations.

These findings affirm that SERS-active Ag_y_FTO substrates can be rapidly and efficiently prepared via electroplating Ag onto FTO substrates. Moreover, using adhesive tape to delineate the electroplating area effectively overcomes the limitations of fixed-size electroplating on FTO substrates.

### 3.2. Photo-Reduction of X-Ag and Characterization

**Figure 6:**
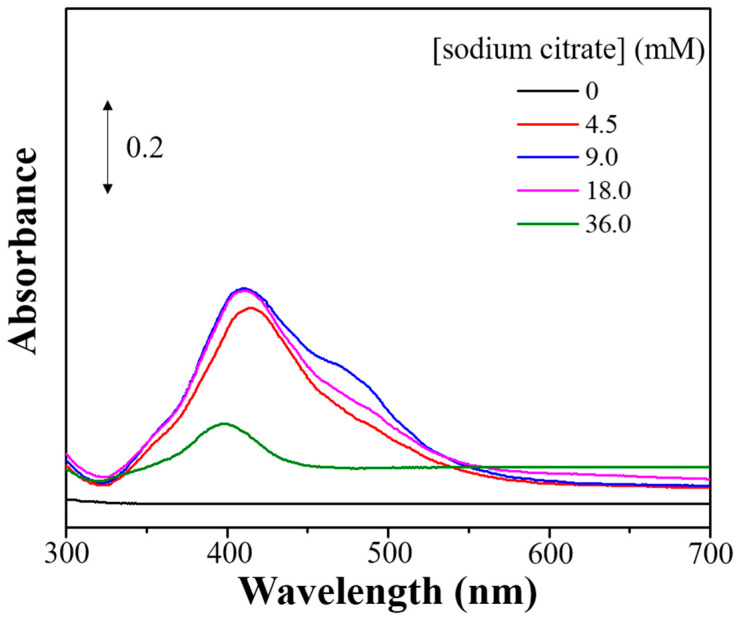
UV–Vis spectra of B-Ag solutions prepared at different concentrations of sodium citrate.

To investigate the enhanced Raman effect by incorporating nanoparticles into the substrate, Ag nanoparticles were produced via photo-reduction with the help of sodium citrate as a reducing agent at room temperature. The influence of the reagent concentration was examined, as illustrated in Figure 6. As a control experiment, no Ag nanoparticle was produced from a solution containing Ag^+^ ions without a reducing agent after exposing it to blue LED light for 30 min. Thus, no absorbance peak was observed in the UV–Vis analysis (black spectrum in Figure 6). On the other hand, introducing 4.5 mM sodium citrate to Ag^+^ solution and exposing it to 10 W blue LED light for 30 min produced the SPR absorption peak at 418 nm in the UV–Vis analysis (red spectrum in Figure 6), indicative of Ag nanoparticles present in the solution, denoted as B-Ag nanoparticles. Increasing the sodium citrate concentration to 9.0 mM and 18.0 mM maximized the SPR absorption peak intensity at 411 nm, with additional satellite signal peaks at 475 nm (blue and pink spectra in Figure 6) observed after exposing the solution to blue LED light for 30 min. Furthermore, an increase in sodium citrate concentration to 36.0 mM led to the persistence of the SPR peak solely at 398 nm but with diminished absorbance (green spectrum in Figure 6). High concentrations of sodium citrate promote the generation of smaller AgNPs (blue-shift). However, due to insufficient illumination reaction time, the overall number of particles is reduced, leading to a decrease in absorbance at 398 nm. To achieve a higher absorbance at 411 nm, a 9.0 mM concentration of sodium citrate was chosen to investigate the effect of LED wavelength and irradiation time in the next study.

**Figure 7:**
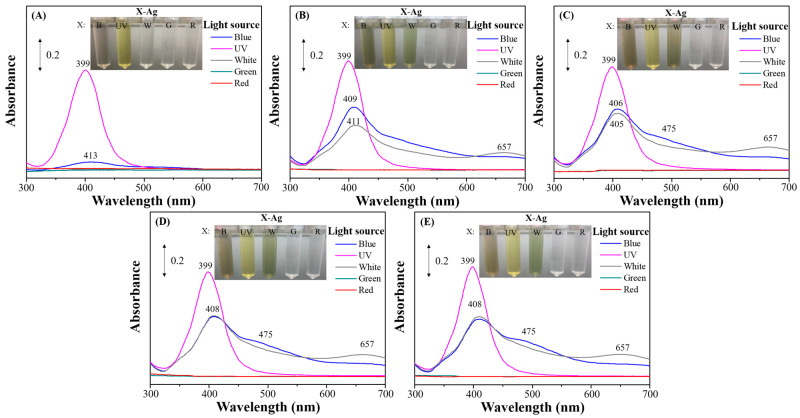
UV–Vis spectra of X-Ag solutions prepared with varying irradiation times: (**A**) 30, (**B**) 60, (**C**) 90, (**D**) 120, and (**E**) 150 min. Inset: photographic images of respective X-Ag solutions.

LED wavelength and irradiation time can be important factors for the light-induced growth of Ag nanoparticles. As depicted in Figure 7, the observable characteristic absorption peaks in UV–Vis spectra reveal that X-Ag nanoparticles are formed using blue LED (B-Ag), UV LED (UV-Ag), and white LED (W-Ag) lights (10 W each). In contrast, irradiation with red and green LED lights (10 W each) produced no X-Ag particle, as evidenced by the lack of characteristic absorbance peaks (red and green spectra in Figure 7A–E). In this study, Ag nanoparticles were not formed by irradiation with green LED light, whereas they were formed by irradiation with an Nd/YAG laser (λ = 532 nm, 10 mW) [33]. Kitahama et al. proposed that Ag nanoparticles could be fabricated at the aperture of an optical fiber (300 nm in diameter, throughput: ∼5%) for a scanning near-field optical microscope. This fabrication occurs via photo-reduction due to the evanescent field, which enhances the near-field signal by plasmon resonance. Different effects of applied LED light sources were observed. Applying UV-LED light to the solution for 30 min produced a yellow solution containing UV-Ag nanoparticles as depicted in the inset of Figure 7A. The absorbance of UV-Ag nanoparticles was the highest among all other applied wavelengths and it stabilized after 30 min, indicating the fastest rate of crystal growth. This can be attributed to the UV light’s wavelength band (365 ± 5 nm) being closely aligned with the SPR characteristic peak of UV-Ag particles (399 nm, evidenced in the pink spectrum of Figure 7A). The second fastest growth rate was B-Ag nanoparticles, which produced an absorption peak at 413 nm after 30 min of blue light exposure. Longer blue light exposure induced a blue shift of the characteristic peak to 409 nm, increased absorbance between 450 and 600 nm, and transitioned the solution color to a deeper amber, as illustrated in Figure 7B. The UV–Vis spectrum of B-Ag nanoparticles revealed a higher pronounced characteristic peak height at 406 nm and 475 nm at 90 min of light exposure, which plateaued after 90 min, confirming the increased formation of B-Ag nanoparticles (blue spectrum in Figure 7C). A minimum irradiation duration of 60 min under white LED light was required to synthesize W-Ag nanoparticles, as evidenced by their SPR characteristic peaks at 411 nm and 657 nm (observed in the gray spectrum of Figure 7B), which continuously increased until they plateaued at 120 min (illustrated in the gray spectra of Figure 7C–E). Interestingly, the resulting W-Ag solution appeared to have a green hue. The results demonstrated that the selection of light wavelength and irradiation duration can influence the size and characteristics of X-Ag nanoparticles [34,35].

**Figure 8:**
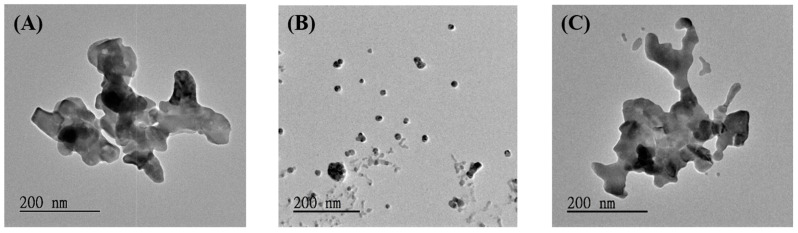
TEM images of (**A**) B-Ag, (**B**) UV-Ag, and (**C**) W-Ag.

To learn more about the effect of LED light on X-Ag nanoparticle growth, the morphology of the synthesized B-Ag, UV-Ag, and W-Ag nanoparticles was analyzed using TEM. The TEM images presented in Figure 8 were taken following 90 min of exposure of the solution containing 9.0 mM sodium citrate and 0.1 mM AgNO_3_ to blue, UV, and white LED light. The images revealed that B-Ag and W-Ag particles exhibited irregular, synapse-like shapes with wide size ranges. In contrast, UV-Ag particles were spherical, averaging 18.3 ± 2.3 nm in diameter. The TEM observations suggest that the unique absorption peaks at 475 nm and 657 nm in the B-Ag and W-Ag spectra may originate from localized surface plasmon resonance (LSPR) effects attributed to the irregular surface topographies of these particles. Meanwhile, the spherical morphology of UV-Ag corresponded with that of traditional synthesis methods, as reflected in their absorption spectrum, aligning with the SPR band of Ag nanoparticles fabricated by traditional approaches. The surface charges of B-Ag, UV-Ag, and W-Ag nanoparticles were analyzed using zeta potential measurements. The results revealed that all three X-Ag nanoparticle variants exhibited negative charges, primarily attributed to the adsorption of negatively charged citrate ions on the surface of the X-Ag nanoparticles as the reason for stabilizing X-Ag nanoparticles in solution. Consequently, this study highlights the dual role of sodium citrate not only as a reducing agent but also as an effective stabilizer for X-Ag nanoparticles.

### 3.3. SERS Performances of X-Ag-Ag_y_FTO Substrates and MG Detection

**Figure 9:**
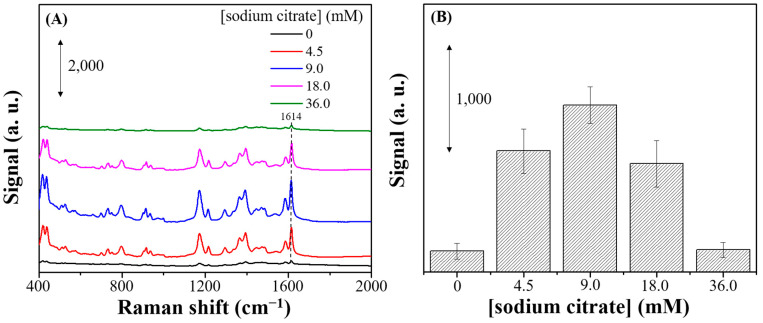
(**A**) SERS spectra and (**B**) Raman signals of MG (1.0 μM) at 1614 cm^−1^ on B-Ag/Ag_1_FTO substrates, where B-Ag was prepared at different concentrations of sodium citrate.

With the interesting irregular shape of B-Ag nanoparticles, further study was conducted to measure its SERS performance on MG detection. Initially, various concentrations of sodium citrate were used to synthesize B-Ag nanoparticles from 90 min of blue LED exposure. These concentrated B-Ag nanoparticles were subsequently applied to the Ag_1_FTO substrate, and the Raman spectroscopic analysis of 1.0 µM MG was conducted, as depicted in Figure 9A. As illustrated in Figure 9B, there was a progressive increase in Raman intensity (at 1614 cm^−1^) correlating with increasing sodium citrate concentrations. The Raman peak intensity of MG maximized at a sodium citrate concentration of 9.0 mM is shown in Figure 9. The B-Ag nanoparticles synthesized at this optimal concentration exhibited a pronounced absorption peak and a distinct characteristic peak at 475 nm, indicating a significant quantity of B-Ag nanoparticles for maximum Raman signal enhancement. Conversely, sodium citrate concentrations deviating from 9.0 mM resulted in Raman signal intensities of MG that were comparable to or lower than those observed using the native Ag_1_FTO substrate, highlighting the critical influence of B-Ag nanoparticles prepared at different sodium citrate concentrations on the efficacy of Raman enhancement.

**Figure 10:**
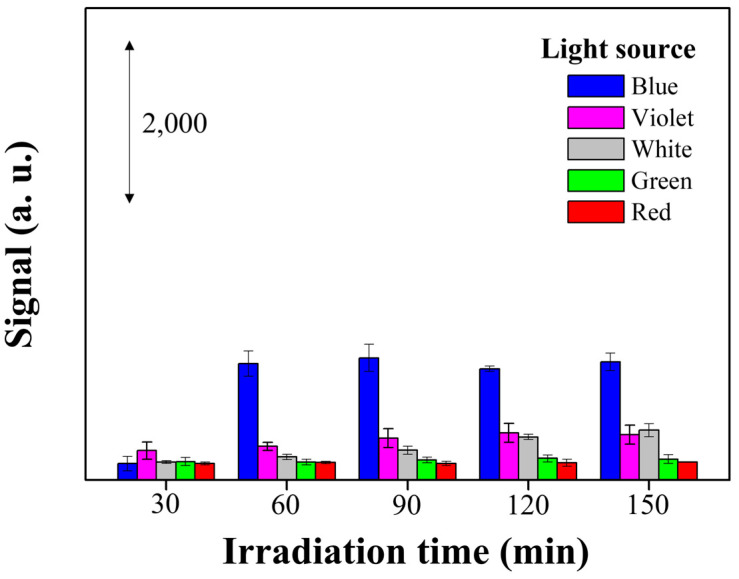
Raman signals of 1.0 μM MG at 1614 cm^−1^ on X-Ag/Ag_1_FTO substrate, where X-Ag was prepared using different light sources.

Subsequently, the influence of different light sources and durations on Raman signal enhancement was assessed, as illustrated in Figure 10. Consistent with previous results, green and red LEDs did not produce particles. No Raman enhancement in the MG signal was observed using the X-Ag-Ag_1_FTO substrates (X: G and R). UV-Ag nanoparticles grown within 30 min using the UV-Ag-Ag_1_FTO substrate produced SERS signals from MG, as shown in Figure 10. Likewise, B-Ag, UV-Ag, and W-Ag nanoparticles grown after 60 min of light exposure also resulted in SERS signals on MG when used as the X-Ag-Ag_1_FTO substrate (X: UV, B, or W), as shown in Figure 10. Notably, when irregularly shaped B-Ag nanoparticles were coupled with the dendritic nature of the Ag_1_FTO substrate, they generated an increased number of hot spots, thereby significantly amplifying the Raman signal, achieving a signal approximately 3.46–6.94 times greater than that of other X-Ag-Ag_1_FTO substrates (X: UV, W, G, and R). UV-Ag nanoparticles are characterized by their spherical shape, resulting in a comparatively modest Raman enhancement effect. Even though W-Ag nanoparticles also exhibited an irregular form, they had a less pronounced Raman enhancement. However, the Raman enhancement associated with W-Ag gradually increased, reaching a plateau after 120 min of irradiation, as depicted by the extended blue bar in Figure 10. In this study, 90 min of blue LED irradiation was considered the optimal condition for synthesizing B-Ag nanoparticles, which produced effective Raman enhancement for MG detection.

**Figure 11:**
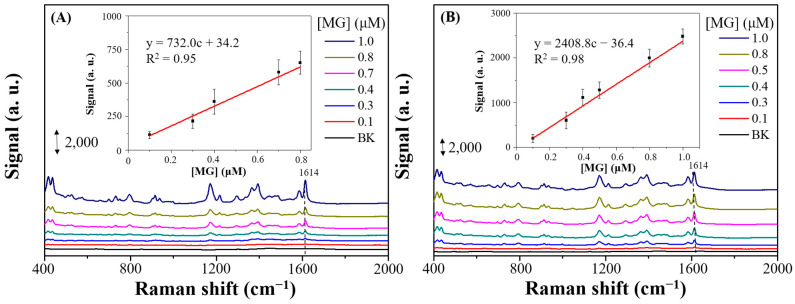
SERS spectra (Inset: Raman signal at 1614 cm^−1^) of MG at different concentrations, analyzed using (**A**) B-Ag/Ag_1_FTO and (**B**) B-Ag/Ag_2_FTO substrates.

From the Raman measurements, Raman EF values were calculated and tabulated in Table 1. The highest was 2.56 × 10^5^ from the B-Ag-Ag_1_FTO substrate, which was selected for quantitative analysis of MG. Different concentrations of MG were applied to the Ag_1_FTO substrate, followed by the addition of concentrated B-Ag nanoparticles to evaluate the analytical calibration line, as shown in Figure 11A. The findings indicate an increase in Raman signal intensity proportional to rising MG concentrations. The linear detection ranges for MG in the B-Ag-Ag_1_FTO configuration span from 0.1 µM to 0.8 µM, with an LOD of 0.07 µM, an S/N ratio of 3.0 ($LOD=\frac{3\sigma_{blank}}{slope}=\frac{3\times 17.1}{732.0}=0.07\;\mu M$), and an R^2^ value of 0.92, as shown in Table 1. Different concentrations of MG were analyzed using the B-Ag-Ag_2_FTO substrate via Raman spectroscopy, and their spectra are depicted in Figure 11B. The results showed a linear detection range from 0.1 µM to 1.0 µM, an LOD of 0.02 µM, an S/N ratio of 3.0 ($LOD=\frac{3\sigma_{blank}}{slope}=\frac{3\times 16.1}{2408.8}=0.02\;\mu M$), and an R^2^ value of 0.98, as documented in Table 1. Upon evaluating sensitivity, the analysis revealed that the B-Ag-Ag_2_FTO system enhanced sensitivity by a factor of 26 compared to Ag_1_FTO. Furthermore, the calculation of the analytic EF value for the B-Ag-Ag_2_FTO combination yielded a value of 2.79 × 10^5^, underscoring the substantial improvement in Raman signal amplification.

**Figure 12:**
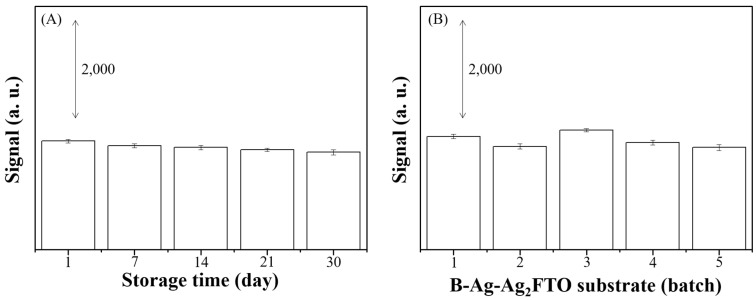
Signal reproducibility of (**A**) different days in B-Ag-Ag_2_FTO substrate and (**B**) different fabricated batches of B-Ag-Ag_2_FTO substrates.

Before testing real samples of aquaculture water, it was crucial to evaluate the signal stability of the B-Ag-Ag_2_FTO substrate over various storage durations and production batches. As demonstrated in Figure 12A, the same batch of B-Ag-Ag_2_FTO substrates was stored in a drying box. After 30 days of storage, the Raman signal retained 90% of its initial intensity. Weekly testing of the same batch of 1 µM MG revealed a relative standard deviation (RSD) not exceeding 2.8%. Figure 12B shows the performance of B-Ag-Ag_2_FTO substrates prepared in different batches, with 1 µM of MG tested. The RSD of the Raman signals from different batches was less than 6.7%. Thus, the B-Ag-Ag_2_FTO substrate developed in this study demonstrated excellent stability.

### 3.4. Determination of MG Concentration in Aquaculture Water Sample Using B-Ag-Ag_2_FTO Substrates

Aquaculture water from the coastal area of Kaohsiung (Taiwan) was used as the matrix material to validate the applicability of the B-Ag-Ag_2_FTO substrate in simulated environment samples. Before experimentation, this sample was centrifuged and filtered with a 0.22 μm microporous membrane. The filtrate was then diluted ten-fold with deionized water, producing a matrix solution for the MG samples. The MG stock solution was spiked into the matrix solution to prepare a diluted seawater solution containing 0.2 to 1.0 μM of MG. These solutions were added to the B-Ag-Ag_2_FTO substrate before the Raman signal was measured after drying.

The spiked recoveries of MG in the aquaculture water samples were between 90.0% and 110.0%, with an RSD of 6.3%, as shown in Table 2. This demonstrates that B-Ag-Ag_2_FTO substrates can be effectively used with an actual environment matrix, transcending the limitations of only utilizing ultrapure water for laboratory detection. They can overcome matrix interferences in real-world water samples, signifying their substantial potential as a practical Raman spectroscopy detection method.

The linear range and LOD of MG in diluted seawater were compiled and compared to the B-Ag-Ag_2_FTO substrate developed, as detailed in Table 3. Although the linear range and LOD of the proposed B-Ag-Ag_2_FTO substrate may not be superior, the methodology employed is notably simpler and quicker, eliminating the extensive time typically required for material synthesis. Additionally, the B-Ag-Ag_2_FTO substrate offers the advantages of cost-effectiveness, minimal sample requirements, and environmental friendliness, making it a promising tool for efficiently detecting MG in various contexts. Looking forward, the B-Ag-Ag_2_FTO process can be applied to flexible substrates, revolutionizing in situ fish sample analysis and bypassing the cumbersome pre-processing steps traditionally required. For future development, we aim to develop a SERS-active, soft substrate using spin-coating or photochemical polymerization to enable direct application to fish skin for rapid testing. This innovative approach, still in development, could significantly streamline and enhance the efficiency of analytical processes in aquaculture applications.

## 4. Conclusions

In this study, electroplating of Ag was successfully employed to fabricate a SERS-active Ag_y_FTO substrate for SERS applications. Through SEM and EDS analyses, the Ag_y_FTO substrate exhibited a unique dendritic structure that amplified the Raman signal due to the electromagnetic field generated within the dendrite structure. The optimized Ag_2_FTO substrate, assessed by SERS analysis of MG, was created from the optimized electroplating conditions with a two-cycle deposition process: the first cycle deposited at 1.0 V for 4 min, followed by a second cycle at 1.0 V for 1 min. Furthermore, SERS enhancement on the Ag_2_FTO substrate was produced by adding B-Ag nanoparticles to the Ag_2_FTO substrate. The combined B-Ag-Ag_2_FTO substrate could detect MG within a concentration range of 0.1 µM to 1.0 µM, with an LOD as low as 0.02 µM. This substrate also demonstrated high recovery rates (90.0% to 110.0%) with an RSD of less than 6.3% in real aquaculture water samples.

## Figures and Tables

**Table 1:** Figure of merit for MG detection using different SERS-active substrates.

Substrate	Linear Range (μM)	R^2^	LOD (μM)	EF Value
Ag_1_FTO	4.0–10.0	0.97	0.52	4.52 × 10^4^
Ag_2_FTO	0.8–10.0	0.97	0.21	6.15 × 10^4^
B-Ag-Ag_1_FTO	0.1–0.8	0.92	0.07	2.56 × 10^5^
B-Ag-Ag_2_FTO	0.1–1.0	0.98	0.02	2.79 × 10^5^

**Table 2:** MG spiked recovery and RSD in aquaculture water samples using B-Ag/Ag_2_FTO substrate.

Spiked (μM)	Detected (μM)	Recovery (%)	RSD (%)
0.20	0.18	90.0	4.3
0.40	0.43	107.5	6.3
0.60	0.63	105.0	5.3
0.80	0.78	97.5	3.9
1.00	1.10	110.0	5.5

**Table 3:** Comparison of MG sensing performance using different SERS-active substrates.

Substrate	Linear Range (M)	LOD (M)	Ref.
Fe_3_O_4_@Au MCS	1.0 × 10^−7^–1.0 × 10^−3^	1.0 × 10^−7^	[21]
Au/cicada wing	1.0 × 10^−7^–1.0 × 10^−3^	1.0 × 10^−7^	[22]
AgNCs	5.0 × 10^−7^–5.0 × 10^−4^	2.6 × 10^−7^	[23]
Paper-based Au/AgNPs	3.9 × 10^−8^–1.0 × 10^−5^	4.3 × 10^−9^	[24]
AuNPs–GO	2.7 × 10^−11^–2.7 × 10^−8^	2.7 × 10^−11^	[25]
B-Ag-Ag_2_FTO	1.0 × 10^−7^–1.0 × 10^−6^	2.0 × 10^−8^	This work

## Data Availability

Data are contained within the article.
